# Comment on Kieslinger et al. A Recurrent *STAT5B^N642H^* Driver Mutation in Feline Alimentary T Cell Lymphoma. *Cancers* 2021, *13*, 5238

**DOI:** 10.3390/cancers14194593

**Published:** 2022-09-22

**Authors:** Valérie Freiche, Lucile Couronné, Julie Bruneau, Olivier Hermine

**Affiliations:** 1 Ecole Nationale Vétérinaire d’Alfort, CHUVA, Unité de Médecine Interne, 94700 Maisons-Alfort, France; 2 Cytogenetics Department, Hôpital Necker—Enfants Malades, Assistance Publique-Hôpitaux de Paris (APHP), Laboratory of Cellular and Molecular Mechanisms of Hematological Disorders and Therapeutical Implications, INSERM U1163, Imagine Institute, University of Paris, 75015 Paris, France; 3 Pathology Department, Hôpital Necker—Enfants Malades, Assistance Publique-Hôpitaux de Paris (APHP), Laboratory of Cellular and Molecular Mechanisms of Hematological Disorders and Therapeutical Implications, INSERM U1163, Imagine Institute, University of Paris, 75015 Paris, France; 4 Hematology Department, Hôpital Necker—Enfants Malades, Assistance Publique-Hôpitaux de Paris (APHP), Laboratory of Cellular and Molecular Mechanisms of Hematological Disorders and Therapeutical Implications, INSERM U1163, Imagine Institute, University of Paris, 75015 Paris, France

We have read with great interest the recently published article by M. Kieslinger et al. in Cancers Journal [1]. In this retrospective study, the authors report the JAK-STAT pathway deregulation in gastrointestinal T-cell lymphoma in cats, and particularly STAT5B broad activation. 

Deregulation of the JAK-STAT pathway has recently emerged as a major oncogenic mechanism in several T & NK leukemia and lymphoma subtypes. This pathway is known to regulate lymphocyte development, differentiation, and proliferation [2]. This article raises the major point of the homology of feline and human STAT3 and STAT5B proteins and the study at a genomic level confirms its strong conservation between species.

We would like to comment on several points the authors made in their article, with regards to the histopathological classification of cases including immunohistochemistry, the comparison of feline intestinal low-grade T-cell lymphoma (LGITL) to human MEITL for Monomorphic Epithelial Intestinal T-cell Lymphoma, and their statement of being first to discover JAK/STAT pathway activation and STAT-5 overexpression in cats with LGITL. 

In the Materials and Methods section, histologic criteria are poorly depicted to describe low-grade gastrointestinal T-cell lymphomas. Furthermore, no immunohistochemistry data is presented to confirm the phenotype of the various cases selected.

Among the different T-cell lymphoma subtypes, feline intestinal low-grade T-cell lymphoma (LGITL) is reported by the authors as a spontaneous model of EATL type II (recently renamed MEITL for Monomorphic Epithelial Intestinal T-cell Lymphoma) [3]. However, the 2016 revision of the World Health Organization classification of lymphoid neoplasms introduced a new entity: the indolent T-cell lymphoproliferative disorder (LPD) of the GI tract (GI-LPDs). Despite feline LGITL and human MEITL show microscopic similarities, their clinical course and their immunophenotyping are dramatically different. Human MEITL neoplasms have an aggressive clinical course with a median survival time of only 7 months. They co-express CD3 and CD56 (a natural killer cell marker), display a high mitotic index with a high expression rate of Ki-67, and do not show concurrent inflammatory lesions [3]. In contrast, feline LGITLs are indolent neoplasms, and they are known to progress slowly. LGITL show a low Ki-67 expression and are most frequently associated with inflammatory lesions. Their phenotype shows CD3+/CD56-. Finally, in a recent prospective study published in June 2021, our team already confirmed the expression of STAT5 in LGITL and validated the feline disease as a relevant spontaneous model for human GI-TLPDs [4]. 
